# Synergetic effect of metal nickel and graphene as a cocatalyst for enhanced photocatalytic hydrogen evolution via dye sensitization

**DOI:** 10.1038/srep10589

**Published:** 2015-06-12

**Authors:** Weiying Zhang, Yuexiang Li, Xianping Zeng, Shaoqin Peng

**Affiliations:** 1 Department of Chemistry, Nanchang University, Nanchang 330031, China

## Abstract

Exploiting new, low-cost and efficient electrocatalysts for hydrogen evolution reaction (HER) is important to resolve the energy crisis and environment pollution. In this work, graphene decorated with Ni nanoparticles (NPs) were synthesized via one-pot reduction using graphene oxide (GO, the obtained composite was denoted as GN) as a precursor. The as-prepared composite GN exhibits much better electrocatalytic and dye-sensitized HER activities than single Ni and reduced graphene oxide (RGO), namely, a great synergetic effect of RGO and Ni for HER. The coupling of metal Ni with the defect carbons of RGO plays a key role in the synergetic effect. The structure of GN composites is another key factor to the synergetic effect. The highest apparent quantum yield (AQY) for dye-sensitized photocatalytic hydrogen evolution at 470 nm reaches 30.3% under the optimal conditions.

Today, the global energy crisis and environment pollution are becoming more and more serious. Developing a new, efficient and clean energy source is extremely urgent. Conversion of solar energy into hydrogen is the most promising strategy. The dye-sensitized photocatalytic hydrogen production from water splitting, essentially simulating the primary processes of photosynthesis^1^, is considered as a facile and effective approach for utilizing solar light to produce storable and clean fuel^2^,^3^,^4^,^5^. In the sensitization system, besides a dye and sensitized matrix^6^,^7^,^8^,^9^, a HER cocatalyst is essential. In general, noble metal Pt is an excellent HER cocatalyst due to its low overpotential^10^. However, Pt is rare and expensive, which hinders the large-scale application. Hence, it is necessary to exploit resource-rich and low-cost materials as the cocatalysts to replace Pt.

Nickel, one of earth-abundant elements, has been received growing attention owing to its electrochemical stability and low cost^11^,^12^. Nevertheless, it is well known that metal Ni locates on the left side of the Volcano plots for HER, because the formation energy of Ni-H is lower than that of Pt-H^13^. As a result, metal Ni as a HER catalyst is less active than Pt.

Graphene, an atom-thick sheet of sp^2^-hybridized carbons, possesses interesting properties, in particular, excellent electron transfer ability and large specific surface area^14^. Because graphene oxide (GO) can be easily prepared by simple and scalable chemical oxidation approaches^15^, the reduction of GO into RGO is a widely-used method to obtain graphene. RGO is used as a support to anchor photocatalysts because it can improve the charge separation and transfer of the photocatalysts, which efficiently enhances the performance of the graphene-based photocatalysts^16^,^17^,^18^.

RGO itself acts as a HER cocatalyst when it was loaded on semiconductors^19^, but its activity is low. Zheng *et al.*^20^ reported that nitrogen-doped graphene (N-graphene) coupled with graphitic-carbon nitride showed much higher HER activity than that of single N-graphene. The enhanced activity stems from their chemical and electronic coupling. This inspires us to couple metal Ni to graphene as an efficient and cheap HER electrocatalyst/cocatalyst.

Due to the oxygen-containing functional groups, GO can bind metal ions and be dispersed well in an aqueous solution^21^. GO is a cheap and ease-obtained precursor to prepare metal-graphene (RGO) hybrids. There are carbon defects in RGO, including defect carbons with sp^2^ dangling bonds which come from the reduction treatment of GO^22^,^23^. The defect carbons enhance the coupling intensity of RGO to metals (chemical coupling). When Ni clusters are loaded on RGO, there are at least two coupling carbon states: carbidic (Ni-C bond) and graphitic, respectively. The carbidic carbons are catalytically active^24^ and H atom adsorption sites for hydrogen storage^25^, while graphitic carbons are inert.

Herein, RGO decorated with Ni NPs were synthesized via one-pot reduction using GO (the composite was denoted as GN) and RGO (the composite as rGN) as precursors, respectively. The obtained GN composites exhibit much better electrocatalytic and dye-sensitized HER activities than single Ni and RGO, respectively. The synergetic effect comes from the coupling of metal Ni with defect carbons of RGO. To the best of our knowledge, this is the first report on the synergetic effect. The activity of GN composite increases by about 3 times compared to that of rGN composite. The findings provide new insights to develop metal-graphene-based electrocatalysts**/**cocatalysts for HER.

## Results

### Performance of GN composites

**Figure 1:**
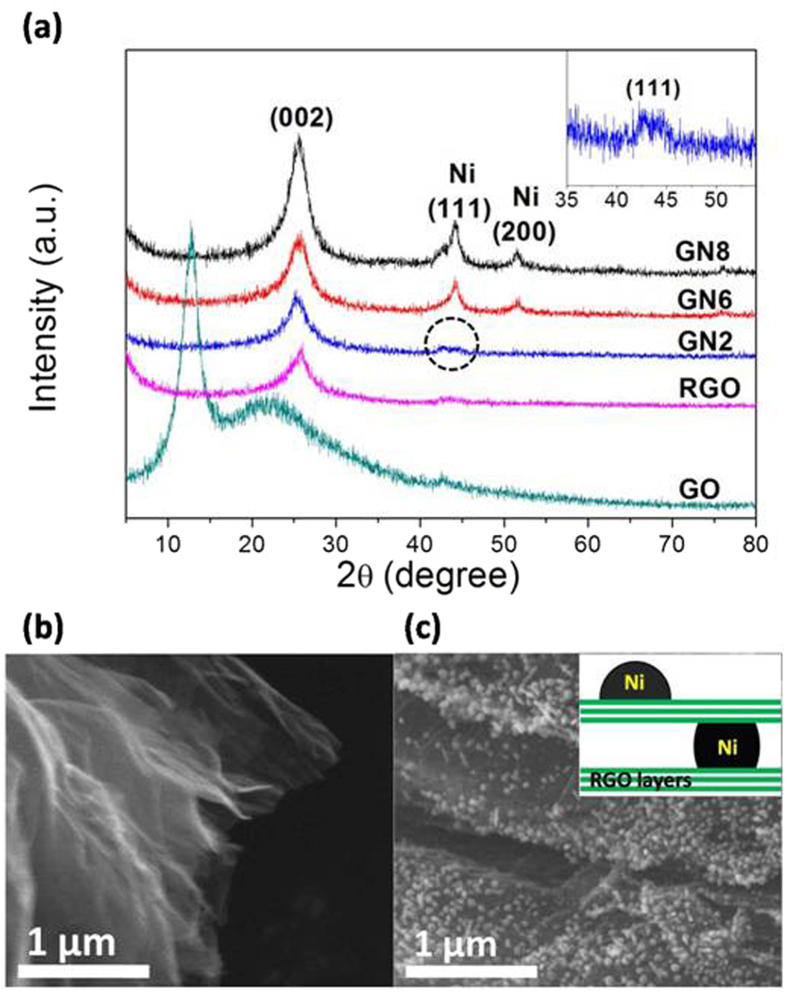
(a) XRD patterns of GO, RGO, GN2, GN6, and GN8. Inset is the enlargement of the selective area for GN2. **(b,c) SEM images of RGO (b) and GN6 composite (c)**. Inset of (**c**) is the schematic illustration of Ni NPs on the exposed RGO surface and in the sandwich structure.

Figure 1a shows the XRD patterns of GO, RGO, GN2, GN6, and GN8. The characteristic peak of GO occurs at 2θ of 12.9°, which corresponds to an interlayer spacing of 0.69 nm (calculated via the Bragg equation), indicating the presence of abundant oxygen-containing groups on both sides of GO sheets^26^. For RGO, GN2, GN6 and GN8, due to thermal treatment in H_2_ ambience, their (002) characteristic peaks shift to around 25.4°, that is, the interlayer spacing decreases to 0.35 nm. This demonstrates that GO is well reduced to graphene (RGO) for single RGO and the composites. Simultaneously, for GN6 and GN8 hybrids, the diffraction peaks at 44.3° and 51.8° assigned to (111) and (200) planes of metallic nickel crystals [JCPDS-NO.04-0783], can be observed clearly. The peaks for GN2 are too weak to be observed fairly. However, as shown in the inset, there is a small distinct peak of (111) facet for metal Ni, confirming that Ni^2+^ is also reduced to metal Ni for GN2.

The average crystallite sizes of metal Ni of GN2, GN6, GN8 and rGN6 (Supplementary Fig. S1) were calculated using the (111) plane diffraction peak (Supplementary Table S1). Compared to the size of Ni NPs for rGN6 (17 nm), the size for GN6 (6.0 nm) is very small. This can be ascribed to the strong interaction of the carbon-vacancy defects of RGO with the metal Ni NPs (Ni-C bond)^27^. Because the defect content of oxygen-containing functional groups for GO as a precursor is much higher than that of RGO, more Ni^2+^ ions can be chemically adsorbed at the defects of GO than at those of RGO. During the thermal reduction in the H_2_ atmosphere, the defects can transform effectively into the carbon-vacancy defects due to the stabilizing effect of the reduced metal Ni clusters via forming Ni-C σ bonds^28^. As a result, more Ni-C bonds are formed for GN6 than for rGN6. Certainly, the exposed part of GO (without Ni clusters) was simultaneously reduced into RGO in this process. Metal Ni clusters are adsorbed weakly on pristine graphene due to relative inertness of sp^2^ bonded carbons^29^. Thus, Ni clusters can diffuse easily along the graphene surface at higher temperature (500 °C) due to less Ni-C bonds, leading quick growth of Ni NPs for rGN6, while sizes of Ni NPs for GN2 (2.8 nm), GN6, and GN8 (7.1 nm) are relatively small since there are more strong Ni-C bonds. Because of preferential chemical adsorption of Ni^2+^ ions on the defects of the precursor GO, the size of the formed Ni NPs for GN2 is very smaller, while those for GN6 and GN8 are larger due to saturation adsorption of Ni^2+^ ions on the defects.

Figure 1b,c display SEM images of RGO and GN6. We can clearly observe Ni NPs exposed on graphene sheets of GN6. For RGO, the graphene sheets are thin and non-stacked, while for GN6 the sheets are thick and well stacked. This demonstrates that the formed Ni NPs could link two or more graphene sheets through forming Ni-C σ bonds, leading to formation of multiple-layer structure (sandwich structure, see the inset of Fig. 1c). As shown in Supplementary Table S1, the specific surface area of RGO is much larger than those of GN2, GN6 and GN8, suggesting that the sandwich structure exists in the three samples. With increasing the Ni content, their specific surface area decreases. The intensity of the (002) peak of GN8 is much stronger than those of GN2 and GN6 (Fig. 1). These indicate that as the Ni content increases, the layer number of the sandwich structure increases, following the order of GN8 > GN6 > GN2. On the other hand, the calculated average thickness of RGO layers for the GN composites slightly enhances with increasing the Ni content (Supplementary Table S1). Moreover, the thickness of rGN6 is larger than that of GN6.

Compared to the Ni NPs on the exposed RGO surface (the inset of Fig. 1c), those in the sandwich structure can couple more carbon-vacancy defects. The more the layer number of the sandwich structure is, the more the coupled defects exist (namely GN8 > GN6 > GN2).

**Figure 2:**
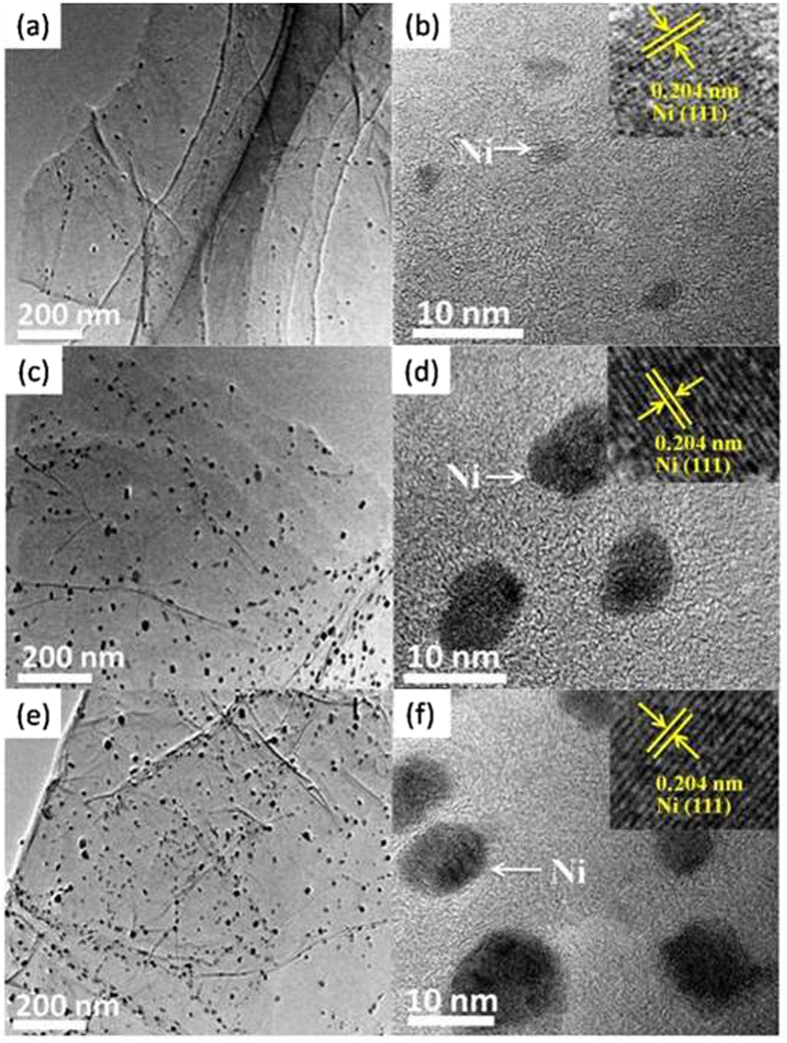
TEM and HRTEM images of GN2 (a, b) GN6 (c, d) and GN8 (e, f).

The morphology and microstructure of the samples were observed using transmission electron microscopy (TEM). Figure 2(a,c,e) indicates that Ni NPs are dispersed highly and uniformly on RGO sheets. This can be attributed to the fact that there are a large amount of carbon defects on GO which stabilize Ni NPs and prohibit their growth. As shown in Fig. 2(b,d,f), the sizes of Ni NPs for GN2, GN6, and GN8 are 2.0-6.4, 4.5-10, and 4.9-12 nm respectively, while the size of rGN6 (Supplementary Fig. S2) is 11-30 nm, which is consistent with the results obtained from XRD (Supplementary Table S1). Moreover, as the amount of deposited Ni increases, the coverage of Ni NPs on RGO sheets (GN2, GN6, and GN8) enhances, resulting in forming more Ni-C σ bonds. As shown in the insets (b, d and f), the lattice spacing of Ni NPs for the three samples is 0.204 nm, which is indexed to the (111) facet of a face-centered cubic Ni phase^30^, confirming further the presence of metal Ni.

**Figure 3:**
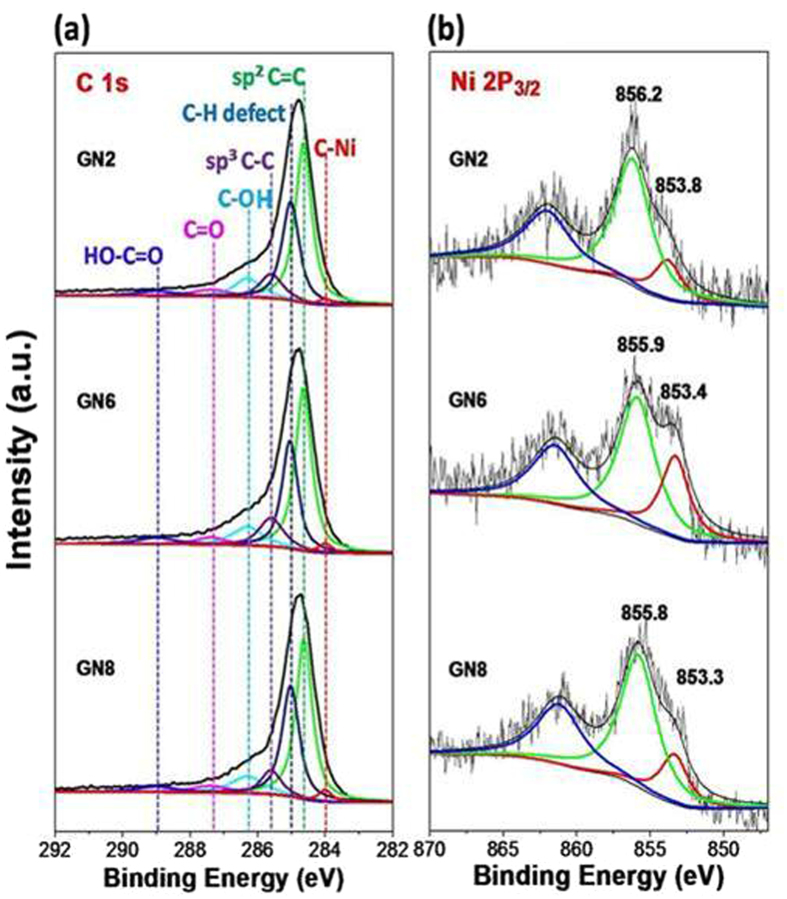
High resolution XPS spectra of C 1s (a), and Ni 2p_3/2_ (b) for GN2, GN6, and GN8.

Figure 3 gives the high resolution XPS spectra of C 1 s and Ni 2p_3/2_ for GN2, GN6, and GN8. For the C 1 s spectra of the three GN composites, the peak intensities of the carbons with oxygen-containing groups (HO-C = O, C = O and C-OH) are very low, while those of C = C are considerably high, indicating the effective reduction of the three composites^31^. There is a very small fitting peak located at 284.0 eV which is assigned to the carbon of carbide nickel^32^, demonstrating the formation of C-Ni bond. The peak intensities for GN2, GN6 and GN8 increase in turn, confirming that the contents of their carbon defects coupled with the covered Ni NPs enhance accordingly due to the increase of the Ni coverage and layer number of the sandwich structure (Figs. 2,3). For rGN6, the intensity of the carbon peak at 284.0 eV is much lower than that of GN6, indicating that RGO as the precursor affords less defect carbons than GO (Supplementary Fig. S3).

The high resolution Ni 2p_3/2_ spectra for GN2, GN6, and GN8 can be deconvoluted into two peaks, respectively. The peak in range of 853.8-853.3 eV can be assigned to the binding energy of the 2p_3/2_ for metal Ni^33^, while the peak occurred in 856.2-855.8 eV corresponds to that for NiO^34^. The formation of NiO is owing to the oxidation of metal Ni NPs under air atmosphere^21^. Compared to the binding energy of the pure metal Ni at 852.8 eV^35^, those of GN2 (853.8 eV), GN6 (853.4 eV), and GN8 (853.3 eV) increase, which can be attributed to the fact that the strong interaction between the defect carbons of RGO and Ni NPs. Why are the binding energies of the three samples different? XPS probes mainly surface atoms of the exposed Ni NPs of the samples (instead of those in inner of the sandwich). The strong interaction exists only at interface between the defect carbons of RGO and Ni NPs. As the size of Ni NPs increases, the Ni atom layers enhance, leading to a great decrease in the binding energies of GN6 and GN8.

### Electrochemical measurements

**Figure 4:**
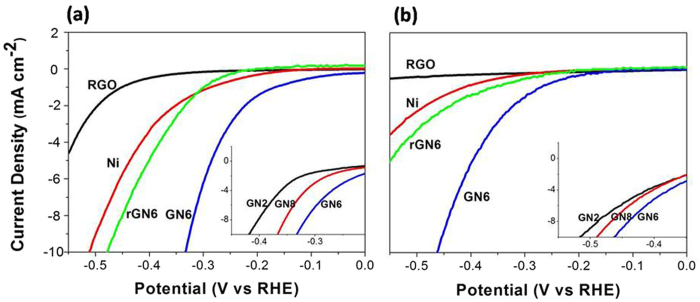
Linear sweep voltammetry (LSV) curves of RGO, Ni, rGN6 and GN6 in 0.50 M H_2_SO_4_ **solution (a) and in 0.50 M Na**_2_SO_4_ solution of pH 10 (b). The insets are LSV curves of GN2, GN6, and GN8 composites.

Figure 4 displays LSV curves of RGO, Ni, rGN6 and GN hybrids under acid condition (a) and basic condition (b). Although H^+^ can be reduced at bare glass carbon electrode in acidic solution among the negative potential range, a control experiment confirms that RGO exhibits HER activity (Supplementary Fig. S4). As shown in Fig. 4a, when the current density for RGO alone is less than 5.0 mA cm^−2^, the overpotential reaches about −0.55 V, suggesting that RGO is a very poor HER electrocatalyst. The overpotential of Ni is around −0.51 V at the current density of 10 mA cm^−2^. Interestingly, GN6 composite exhibits a low overpotential (about −0.33 V) at 10 mA cm^−2^, much lower than those of RGO alone and Ni alone. This confirms that the GN6 composite exhibits a great synergetic effect for HER. However, the overpotential of −0.47 V for rGN6 composite is much higher than that for GN6. This means that the precursor (GO and RGO) performances of GN and rGN composites remarkably affect the HER activities. Moreover, the inset of Fig. 4a clearly shows the HER efficiency is influenced by the Ni loading content on RGO sheets, and the order of the overpotentials is GN2 > GN8 > GN6. GN6 exhibits the highest HER activity.

Under basic condition, as shown in Fig. 4b, the order of the electrocatalytic HER activities for RGO, Ni, rGN6, and GN hybrids remains unchanged compared to that under acid condition. However, overall, their electrocatalytic HER activities decrease slightly. The surface oxidation of the Ni NPs for the GN composites does not obviously affect the HER performance of the composites (Supplementary Fig. S5). Under the basic condition, the durability of GN6 composite has been tested (Supplementary Fig. S6). Surprisingly, the activity of GN6 increases steadily after 100 and 200 potential cycles, which may be due to further reduction of RGO. The deep investigation is underway.

The linear portions of the Tafel plots are fitted by the Tafel equation (η = b log j + a, where j is the current density, and b is the Tafel slope), and the slopes are listed in Table 1. Under acid condition, the Tafel slope of metallic Ni is about 110 mV dec^−1^, indicating that the adsorption of H atoms at Ni surface is a rate-determining step for HER, which is accordant to previous reports^36^,^37^. RGO alone has the largest Tafel slope value of 131 mV dec^−1^, suggesting that the adsorption of H atom would be very difficult, which results in the lowest HER activity. After the hybridization, the Tafel slopes for GN2, GN6, and GN8 are about 85, 64, and 68 mV dec^−1^ respectively, which does not correspond to any simple kinetic model (Volmer, Heyrovsky or Tafel reaction)^38^, indicating complex mechanisms for hydrogen evolution on the GN hybrids. Furthermore, the Tafel slope for GN2 is higher, while the slope for GN6 is close to that for GN8, probably due to the difference in the interaction between the carbon-vacancy defects and Ni NPs and the difference in their sandwich structures. The Tafel slope of 98 mV dec^−1^ for rGN6 is much higher than that for GN6, confirming further that the differences are key factors. Under alkaline condition, the Tafel slopes display similar results as under acid condition.

### Photocatalytic performance

**Figure 5:**
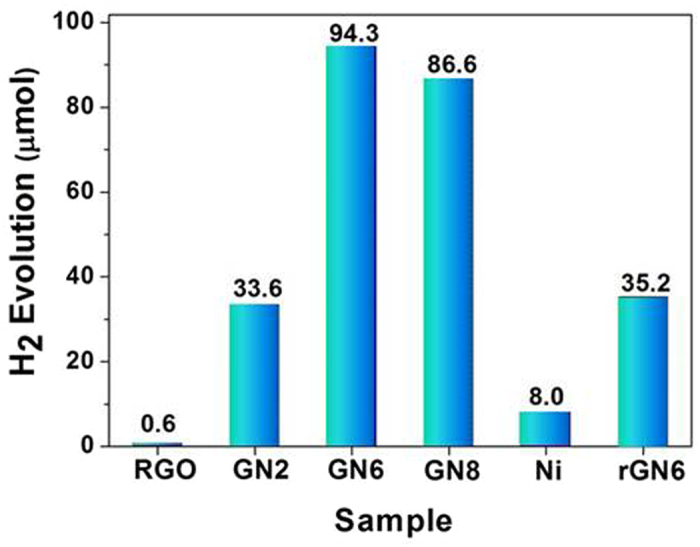
Comparison of the photocatalytic H_2_ evolution activity of RGO, Ni, rGN6, and GN composites. Conditions: 10 mg of the sample; 2.0 × 10^−4^ M Eosin Y (EY); 7.7 × 10^−2^ M trimethylamine (TMA), pH 10; Hg lamp (λ ≥ 420 nm) irradiation 1 h.

Figure 5 shows the dye-sensitized photocatalytic H_2_ evolution activity of RGO, Ni, rGN6, and GN composites. The rate of H_2_ evolution over RGO alone is very low, only 0.60 μmol h^−1^. Although the photocatalytic activity of metal Ni is higher than that of RGO, the efficiency is still low with the rate of 8.0 μmol h^−1^, which indicates that both single RGO and Ni are low active cocatalysts for HER. However, with 2.0 wt% Ni on RGO, the H_2_ evolution rate for GN2 is up to 33.6 μmol h^−1^. When the Ni amount increases to 6.0 wt%, the photocatalytic activity significantly enhances. The H_2_ evolution rate reaches 94.3 μmol h^−1^, corresponding to AQY of 12.5%. However, further increasing the Ni content to 8.0 wt%, the photocatalytic H_2_ evolution activity slightly decreases to 86.6 μmol h^−1^. It is worth noting that the maximum of H_2_ evolution rate of GN6 is 157 and 13 times higher than that of the RGO alone and that of Ni alone, respectively, which means a significant synergetic effect between Ni and RGO for HER. Interestingly, the photocatalytic H_2_ production rate of rGN6 with RGO as the precursor is 35.2 μmol h^−1^, only about one third of GN6.

We also investigated the effect of pH, EY concentration, and the cocatalyst amount on photocatalytic H_2_ activity in EY-GN6 system (Supplementary Fig. S7). For the reaction system, the optimal conditions are pH 10, EY concentration of 3.0×10^−4^ M, and 10 mg of GN6.

**Figure 6:**
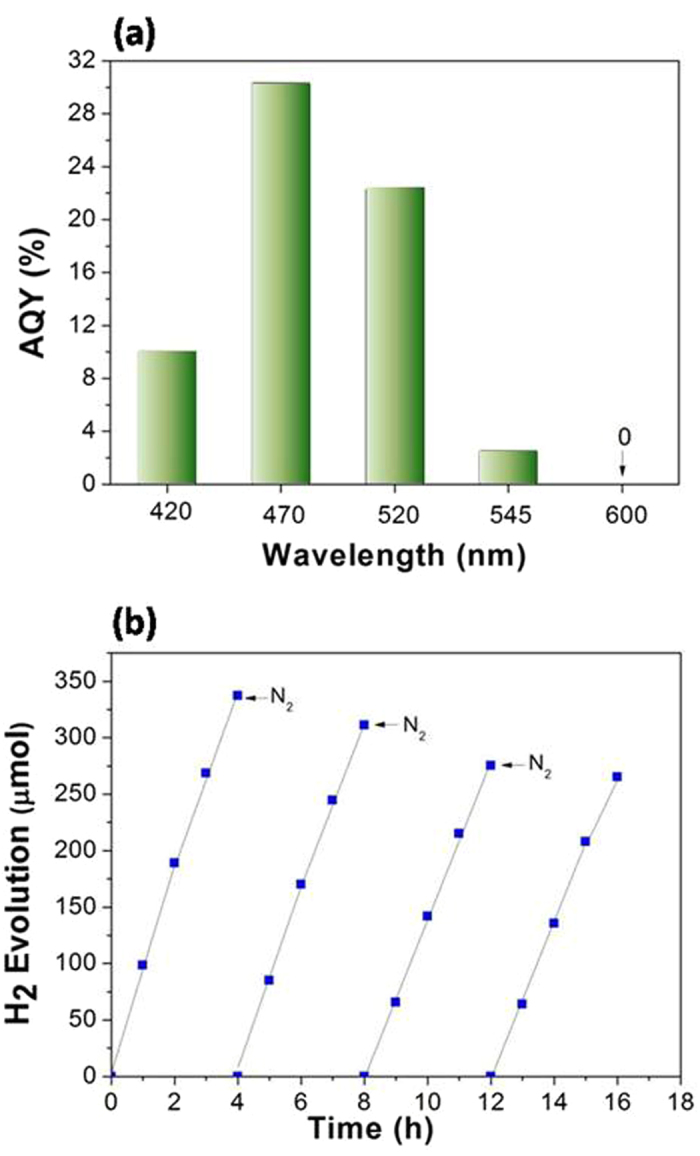
(a) AQY of GN6 cocatalyst plotted as a function of wavelength of the incident light. Conditions: 10 mg of GN6; 3.0 × 10^−4^ M EY; 7.7 × 10^−2^ M TMA, pH 10; LED irradiation 2 h. **(b) Time course of photocatalytic hydrogen evolution in EY-sensitized GN6 reaction system.** After each 4 h irradiation with the Hg lamp, the reacted suspension was filtered and washed with distilled water several times to recover the cocatalyst, and then the obtained cocatalyst was transferred to the reaction cell again. The other procedures are the same as in Fig. 5.

Figure 6a displays the AQY for GN6 at different incident light wavelengths under the optimal conditions. The highest AQY reaches up to 30.3% at 470 nm. Figure 6b shows that in the 4 reaction runs, the GN6 system exhibits stable photocatalytic hydrogen production. The slight reduction of the H_2_ evolution activity is probably attributed to the loss of GN6 in the filtration processes.

## Discussion

**Figure 7:**
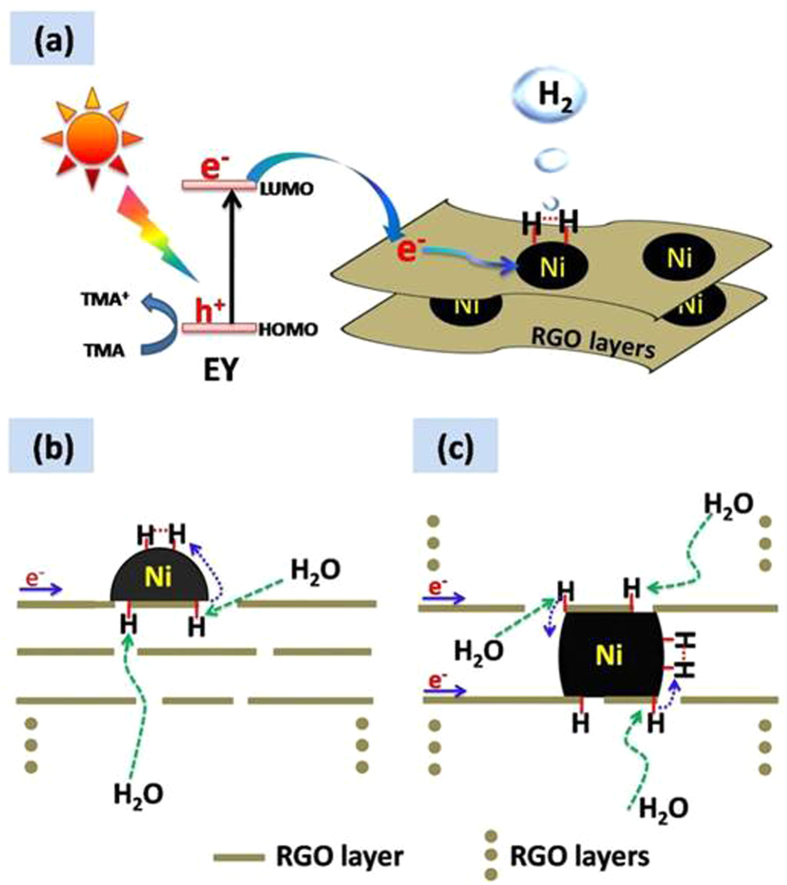
Proposed mechanism for EY-sensitized photocatalytic H_2_ production over GN hybrids under visible light irradiation. H_2_O molecules permeate RGO layer/layers to the coupled defect carbons via defects and/or edges of graphene. The defect carbons are active sites to form H atoms.

Based on the above results, as shown in Fig. 7, the mechanism for the EY-sensitized photocatalytic H_2_ evolution over GN cocatalysts is proposed. When dye EY molecules (LUMO level at −3.45 eV vs vacuum)^39^ are excited under visible light irradiation, the photogenerated electrons can be quickly transferred to lower LUMO level (−4.66 eV vs vacuum)^39^ of RGO sheets. The produced EY^+^ is recovered into EY by the electron donor TMA. These processes can be described by the following reactions.


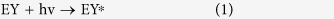



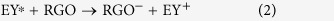



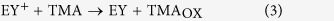


Because of the high conductivity of RGO layer, the injected electrons can effectively transfer along the layer and be trapped by defect carbons (Fig. 7a). The defect carbons act as active sites for hydrogen evolution^40^.


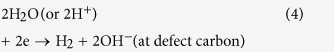


For this process, at first, the reactants H_2_O molecules permeate RGO layer/layers to reach the active sites via the defects or edges of RGO (Fig. 7b,c), and then the H_2_O molecules are reduced into H atoms via forming C-H bond at the defect carbons (active sites) coupled with Ni NPs^41^.


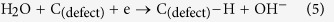


The C-H bond energy (0.82 eV)^42^ of pristine RGO is so weak that the adsorption of the H atoms is unstable and becomes the rate-determined step for HER, leading to the lowest HER activity (Figs. 4,5).

After the coupling with Ni clusters, the C-H bond energy of graphene is increased substantially (by 0.96 -1.15 eV)^42^. As a result, in the GN composites, the H atoms become stable and a large amount of them can exist at the graphene interface covered with Ni NPs, which has been witnessed by Mattia Gaboardi *et al.* via Muon spin relaxation^43^. Owing to very low activation energy barrier of chemisorbed hydrogen (30 meV)^43^, the H atoms adsorbed on the defect carbons can diffuse along the graphene layer to the surface of metal Ni NPs. The binding energy of molecule H_2_ at the coupled metal Ni cluster is relatively low (0.2-1.2 eV), depending on Ni coverage^44^. Thus, H_2_ molecule can be easily desorbed from the Ni surface. As a result, when the formed H atoms diffuse to the Ni surface, they recombine to form H_2_.


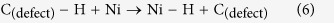



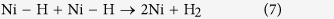


In the two reactions, both RGO (defect carbons) and metal Ni NPs jointly promote HER, namely, producing a synergetic effect. Similarly, the diffused H atoms can also recombine at the surface of the oxidized species NiO or Ni(OH)_2_ to produce H_2_. Thus, GN composites exhibit much higher HER activities than those of single RGO and metal Ni (Figs. 4,5).

After the hybridization, Ni-H bond energy at the interface with graphene should increase due to the strong interaction (Fig. 3 XPS and the calculation^42^). This stabilizes further the H atoms and boosts their diffusion to the surface of Ni NPs. Thus, reaction (6) can take place more effectively due to the coupling.

For the Ni NPs on the exposed sheet, they contact only one of RGO layers (Fig. 7b), while those in the sandwich structure cover the two layers of different RGO sheets (Fig. 7c). Thus, in the multiple-layer structure, the Ni NPs can cover more defect carbons (producing active sites). As the Ni content increases from GN2 to GN6, the enhancement of the layer number of the sandwich structure for the GN composites leads to forming more H atoms, which is advantageous to H_2_ evolution. In addition, because Ni NPs size for GN2 is smaller, the defect carbon content coupled with Ni NPs is lower (Fig. 2), also leading to low HER activity. Thus, the activity of GN2 is much lower than those of GN6 and GN8. However, when Ni NPs size is larger (GN8), they would cover too many defect carbons or edges. Hence, the permeation of reactants H_2_O molecules to the active sites would become difficult owing to long diffuse distance, which is unfavourable to the H atom formation. Further, the thickest RGO layers for GN8 are detrimental to charge transfer due to the thickness effect of RGO layers^18^. As a result, the activity of GN8 is lower than that of GN6.

Valentina Tozzini *et al.* found that RGO containing more defect carbons had much stronger ability to adsorb H atoms than pristine graphene with few defect carbons^45^. The content of carbon-vacancy defect for rGN6 would be much lower than that for GN6. Ni NPs size for rGN6 is so larger (17 nm) that the diffusion of the reactants H_2_O molecules to the active sites becomes very slow. Moreover, the thickness of RGO layers for rGN6 is also larger than that of GN6 (Supplementary Table S1). Thus, the activity of rGN6 is much lower than that of GN6 (Figs. 4, 5).

In summary, the GN composites with various Ni contents are synthesized by one-pot reduction of GO/NiCl_2_ mixture. The GN composites exhibit much better electrocatalytic and dye-sensitized HER activities than RGO alone and Ni alone. The synergetic effect originates from the coupling of Ni NPs with defect carbons of RGO, and the Ni loading content and the structure of the composite are two key factors. GN6 exhibits the highest HER activity. The AQY of GN6 for dye-sensitization hydrogen evolution reaches 30.3% at 470 nm. Moreover, the activity of GN6 composite is about three-fold of rGN6 composite.

## Methods

### Preparation of GN and rGN composites

Except EY (bioreagent), analytical grade reagents were used without further purification. GO was synthesized by the modified Hummers method^14^ using natural flake graphite powder (Sinopharm Chemical Reagent Co. Ltd). A certain volume of NiCl_2_ solution (10 mM) was dropwise added into 200 mL GO solution (1.0 mg mL^−1^) prepared by 30 min ultrasonication, and then kept stirring for 2 h. The water of suspensions was removed in an oven at 80 °C. The obtained GO/NiCl_2_ powders were put into a quartz tube, and treated in reduction ambient [N_2_ (95%) + H_2_ (5%)] at 500 °C for 2 h to obtain the GN composites. The mass ratios of Ni to GO were 2.0, 6.0 and 8.0 (wt%), and the resulting samples were labelled as GN2, GN6, and GN8, respectively. The metallic Ni, RGO and rGN6 composite (RGO/NiCl_2_ as precursor) were also prepared for the purpose of comparison under the same experimental conditions.

### Characterization

X-ray diffraction (XRD) studies were carried out using a XD-2/3 polycrystalline X-ray diffractometer with nickel-filtered Cu Kα radiation as the X-ray source. The transmission electron microscopy (TEM) and High-resolution TEM (HRTEM) images were taken on a JEOL JEM-2100 (kabuskiki kaisha, Japan) equipped with an energy dispersive spectrometer (EDS). For this measurement, the fresh GN and rGN6 composites were immediately dispersed in isopropanol. The scan electron microscopy (SEM) images were taken on a FEI QUANTA 200F. X-ray photoelectron spectra (XPS) were performed on an AMICUS equipped with an Al Kα X-ray source (Japan). Specific surface area tests were measured on DA-BET surface analyzer (JW-DA, China) with N_2_ as adsorbed gas and He as carrier gas.

### Electrochemical measurements

The HER polarization curves of RGO, Ni, rGN6 and GN composite samples were performed on LK98B II electrochemical workstation in a similar manner as described in ref 38. 5.0 mg of the above samples and 50 μL of 5.0 wt% Nafion solution (Dupont, America) were dispersed in 2.0 mL of distilled water by at least 2 h sonication to form a homogeneous ink. Then 10 μL of the catalyst ink (containing 25 μg of catalyst) was loaded onto a glassy carbon electrode of 3 mm in diameter (loading content ~0.35 mg cm^−2^). Linear sweep voltammetry (LSV) with scan rate of 5.0 mV s ^−1^ was conducted in 0.50 M H_2_SO_4_ and 0.50 M Na_2_SO_4_ of pH 10 saturated with N_2_, respectively. A saturated calomel electrode (SCE) was used as the reference electrode, and a Pt wire electrode as the counter electrode. In all measurements, SCE was used as the reference. The measured potentials were converted into the reversible hydrogen electrode (RHE) scale according to the Nernst equation:


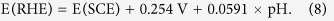


### Photocatalytic performance

The dye-sensitized photocatalytic H_2_ evolution activities were evaluated in a similar method as described in ref 9. The photocatalytic reaction was conducted at room temperature in a 190 mL Pyrex cell with a side flat window (an efficient irradiation area of ca. 16.9 cm^2^). The light source was a 400 W high pressure Hg lamp, equipped with a cutoff filter (λ ≥ 420 nm) and a water cooling jacket to remove UV and the IR illumination. In a typical photocatalytic experiment, a GN composite and 100 mL of aqueous solution containing EY and TMA were added into the cell. Before irradiation, the resulting suspension was treated with sonication for 20 min, and then bubbled with N_2_ through the reaction mixture for 30 min to remove oxygen completely. A silicone rubber septum was fixed at the top of the cell, and the intermittent sampling was conducted through the septum. The amount of photocatalytic hydrogen evolution was determined on a gas chromatograph (TCD as detector, 13X molecular sieve column, N_2_ as gas carrier).

The average incident photon flux was 248.4 μmol m^−2^ s^−1^, which was determined on a FGH-1 Ray virtual radiation actinometer (light spectrum: 400-700 nm). The AQY was calculated via the following equation.


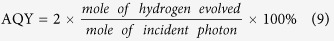


The monochromatic light quantum yields were also measured using various monochromatic LED lamps (UVEC-4, Shenzhen LAMPLIC Science Co. Ltd, China) as the light sources.

## Additional Information

**How to cite this article**: Zhang, W. *et al.* Synergetic effect of metal nickel and graphene as a cocatalyst for enhanced photocatalytic hydrogen evolution via dye sensitization. *Sci. Rep.* **5**, 10589; doi: 10.1038/srep10589 (2015).

## Supplementary Material

Supplementary Information

## Figures and Tables

**Table 1:** The Tafel slopes obtained by the LSV curves from Fig. 4.

**Sample**	**Tafel slope**	
**acid**	**alkaline**	
RGO	131	173
Ni	110	120
GN2	85	90
GN6	64	70
GN8	68	82
rGN6	98	99

## References

[b1] Komaya Y., Miki T., Wang X. F. & Nagae H. Dye-sensitized solar cells based on the principles and materials of photosynthesis: mechanisms of suppression and enhancement of photocurrent and conversion efficiency. Int. J. Mol. Sci. Nov. 10, 4575–4622 (2009). 10.3390/ijms10114575 PMC2808002 20087456

[b2] Youngblood W. J., Lee S. H. A., Maeda K. & Mallouk T. E. Visible light water splitting using dye-sensitized oxide semiconductors. Acc. Chem. Res. 42, 1966–1973 (2009). 19905000 10.1021/ar9002398

[b3] Lazarides T. *et al.* Photocatalytic hydrogen production from a noble metal free system based on a water soluble porphyrin derivative and a cobaloxime catalyst. Chem. Commun. 50, 521–523 (2014). 10.1039/c3cc45025b 23938601

[b4] Zhang P. *et al.* Photochemical H_2_ production with noble-metal-free molecular devices comprising a porphyrin photosensitizer and a cobaloxime catalyst. Chem. Commun. 46, 8806–8808 (2010). 10.1039/c0cc03154b 20957270

[b5] Kong C., Min S. X. & Lu G. X. Dye-sensitized NiS_x_ catalyst decorated on graphene for highly efficient reduction of water to hydrogen under visible light irradiation. ACS Catal. 4, 2763–2769 (2014).

[b6] Li Y. X., Guo M. M., Peng S. Q., Lu G. X. & Li S. B. Formation of multilayer-Eosin Y-sensitized TiO_2_ via Fe^3+^ coupling for efficient visible-light photocatalytic hydrogen evolution. Int. J. Hydrogen Energy 34, 5629–5636 (2009).

[b7] Xu J. Y., Li Y. X., Peng S. Q., Lu G. X. & Li S. B. Eosin Y-sensitized graphitic carbon nitride fabricated by heating urea for visible light photocatalytic hydrogen evolution: the effect of the pyrolysis temperature of urea. Phys. Chem. Chem. Phys. 15, 7657–7665 (2013). 23591628 10.1039/c3cp44687e

[b8] Li Q. Y. & Lu G. X. Visible-light driven photocatalytic hydrogen generation on Eosin Y-sensitized Pt-loaded nanotube Na_2_Ti_2_O_4_(OH)_2_. J. Mol. Catal. A: Chem. 266, 75–79 (2007).

[b9] Zhang W. Y., Li Y. X., Peng S. Q. & Cai X. Enhancement of photocatalytic H_2_ evolution of eosin Y-sensitized reduced graphene oxide through a simple photoreaction. Beilstein J. Nanotechnol. 5, 801–811 (2014). 24991517 10.3762/bjnano.5.92 PMC4077509

[b10] Liu X., Li Y. X., Peng S. Q., Lu G. X. & Li S. B. Photocatalytic hydrogen evolution under visible light irradiation by the polyoxometalate α-[AlSiW_11_(H_2_O)O_39_]^5−^ - Eosin Y system. Int. J. Hydrogen Energy 37, 12150–12157 (2012).

[b11] Merki D., Vrubel H., Rovelli L., Fierro S. & Hu X. L. Fe, Co, and Ni ions promote the catalytic activity of amorphous molybdenum sulfide films for hydrogen evolution. Chem. Sci. 3, 2515–2525 (2012).

[b12] Wang Z. H. *et al.* High electrocatalytic activity of non-noble Ni-Co/graphene catalyst for direct ethanol fuel cells. J. Solid State Electrochem. 17, 99–107 (2013).

[b13] Quaino P., Juarez F., Santos E. & Schmickler W. Volcano plots in hydrogen electrocatalysis - uses and abuses. Beilstein J. Nanotechnol. 5, 846–854 (2014). 24991521 10.3762/bjnano.5.96 PMC4077405

[b14] Geim A. K. Graphene: status and prospects. Science 324, 1530–1534 (2009). 19541989 10.1126/science.1158877

[b15] Chen D., Feng H. B. & Li J. h. Graphene oxide: preparation, functionalization, and electrochemical applications. Chem. Rev. 112, 6027–6053 (2012). 22889102 10.1021/cr300115g

[b16] Li X. *et al.* Engineering heterogeneous semiconductors for solar water splitting. J. Mater. Chem. A 3, 2485–2534 (2015).

[b17] Xiang Q. J. & Yu J. G. Graphene-Based Photocatalysts for Hydrogen Generation. J. Phys. Chem. Lett. 4, 753−759 (2013). 10.1021/jz302048d 26281930

[b18] Aleksandrzak M., Adamski P., Kukułka W., Zielinska B. & Mijowska E. Effect of graphene thickness on photocatalytic activity of TiO_2_-graphene nanocomposites. Appl. Surf. Sci. 331, 193–199 (2015).

[b19] Li Q. *et al.* Highly efficient visible-light-driven photocatalytic hydrogen production of CdS-cluster-decorated graphene nanosheets. J. Am. Chem. Soc. 133, 10878–10884 (2011). 21639097 10.1021/ja2025454

[b20] Zheng Y. *et al.* Hydrogen evolution by a metal-free electrocatalyst. Nat. Commun. 5, 3783 (2014). 24769657 10.1038/ncomms4783

[b21] Ogata C. *et al.* Metal permeation into multi-layered graphene oxide. Sci. Rep. 4, 3647 (2014). 24413270 10.1038/srep03647 PMC3888985

[b22] Hou X. L. *et al.* Tuning radical species in graphene oxide in aqueous solution by photo-irradiation. J. Phys. Chem. C 117, 6788–6793 (2013).

[b23] Gao X. F., Jang J. & Nagase S. Hydrazine and thermal reduction of graphene oxide: reaction mechanisms, product structures, and reaction design. J. Phys. Chem. C 114, 832–842 (2010).

[b24] Fadil N. A. *et al.* Synthesis and electrocatalytic performance of atomically ordered nickel carbide (Ni_3_C) nanoparticles. Chem. Commun. 50, 6451–6453(2014). 10.1039/c4cc01336k 24752450

[b25] Feng Y. Z., Zhen X. J. & Kong S. S. Studies of the mobility of surface carbon formed by the decomposition of methane on supported transition metal catalysts. J. Nat. Gas Chem. 5, 51–58 (1996).

[b26] Pei S. f., Zhao J. P., Du J. H., Ren W. C. & Cheng H. M. Direct reduction of graphene oxide films into highly conductive and flexible graphene films by hydrohalic acids. Carbon 48, 4466–4474 (2010).

[b27] Song W., Jiao M. G., Li K., Wang Y. & Wu Z. J. Theoretical study on the interaction of pristine, defective and strained graphene with Fe_n_ and Ni_n_ (n = 13, 38, 55) clusters. Chem. Phys. Lett. 588, 203–207 (2013).

[b28] Dahal A. & Batzill M. Graphene–nickel interfaces: a review. Nanoscale. 6, 2548–2562 (2014). 24477601 10.1039/c3nr05279f

[b29] Mittendorfer F. *et al.* Graphene on Ni(111): strong interaction and weak adsorption. Phys. Rev. B 84, 201401 (2011).

[b30] Mandal S. & Saha S. K. Ni/graphene/Ni nanostructures for spintronic applications. Nanoscale 4, 986–990 (2012). 22234361 10.1039/c2nr11527a

[b31] Koinuma M. *et al.* Analysis of reduced graphene oxides by x-ray photoelectron spectroscopy and electrochemical capacitance. Chem. Lett. 42, 924–926 (2013).

[b32] Sinharoy S. & Levenson L. L. The formation and decomposition of nickel carbide in evaporated nickel films on graphite. Thin Solid Films 53, 31–36 (1978).

[b33] Grim S. O., Matienzo L. J. & Swartz W. E. X-Ray photoelectron spectroscopy of some nickel dithiolate complexes. J. Am. Chem. Soc. 94, 5116–5117 (1972).

[b34] Ertl G., Hierl R., Knozinger H. & Urbach H. P. XPS study of copper aluminate catalysts. Appl. Surf. Sci. 5, 49–64 (1980).

[b35] Mansour A. N. Nickel monocromated Al Kα XPS spectra from the physical electronics model 5400 spectrometer. Surf. Sci. Spectra 3, 221–230 (1994).

[b36] Martinez S., Huković M. M. & Valek L. Electrocatalytic properties of electrodeposited Ni–15Mo cathodes for the HER in acid solutions: Synergistic electronic effect. J. Mol. Catal. A: Chem. 245, 114–121 (2006).

[b37] Flores E. N., Chong Z. W. & Omanovic S. Characterization of Ni, NiMo, NiW and NiFe electroactive coatings as electrocatalysts for hydrogen evolution in an acidic medium. J. Mol. Catal. A: Chem. 226, 179–197 (2005).

[b38] Li Y. G. *et al.* MoS_2_ nanoparticles grown on graphene: an advanced catalyst for the hydrogen evolution reaction, J. Am. Chem. Soc. 133, 7296–7299 (2011). 21510646 10.1021/ja201269b

[b39] Min S. X. & Lu G. X. Promoted photoinduced charge separation and directional electron transfer over dispersible xanthene dyes sensitized graphene sheets for efficient solar H_2_ evolution. Int. J. Hydrogen Energy 38, 2106–2116 (2013).

[b40] Matsumoto Y. *et al.* Photoreaction of graphene oxide nanosheets in water. J. Phys. Chem. C 115, 19280–19286 (2011).

[b41] Ivanovskaya V. V. *et al.* Hydrogen adsorption on graphene: a first principles study. Eur. Phys. J. B 76, 481–486 (2010).

[b42] Li Q. Z., Wang H. Y., Xia H. P., Wei S. H. & Yang J. H. Density functional study of hydrogen adsorption and diffusion on Ni-loaded graphene and graphene oxide. Int. J. Quantum Chem. 114, 879–884 (2014).

[b43] Gaboardi M. *et al.* Decoration of graphene with nickel nanoparticles: study of the interaction with hydrogen. J. Mater. Chem. A 2, 1039–1046 (2014).

[b44] Wong J., Yadav S., Tam J. & Singh C. V. A van der Waals density functional theory comparison of metal decorated graphene systems for hydrogen adsorption. J. Appl. Phys. 115, 224301 (2014).

[b45] Tozzini V. & Pellegrini V. Prospects for hydrogen storage in graphene. Phys. Chem. Chem. Phys. 15, 80–89 (2013). 23165421 10.1039/c2cp42538f

