# Supplementary material for: Synergetic effect of metal nickel and graphene as a cocatalyst for enhanced photocatalytic hydrogen evolution via dye sensitization
Source: Sci Rep. 2015 Jun 12;5:10589. doi: 10.1038/srep10589 (PMC4464385; doi:10.1038/srep10589)
Supplement: Supplementary Information [file srep10589-s1.pdf]

Supplementary Information

Synergetic effect of metal nickel and graphene as a cocatalyst for enhanced photocatalytic hydrogen evolution via dye sensitization

Weiying Zhang, Yuexiang Li *, Xianping Zeng, Shaoqin Peng

Department of Chemistry, Nanchang University, Nanchang 330031, China.

*Corresponding author. E-mail: Yuexiang Li-liyx@ncu.edu.cn

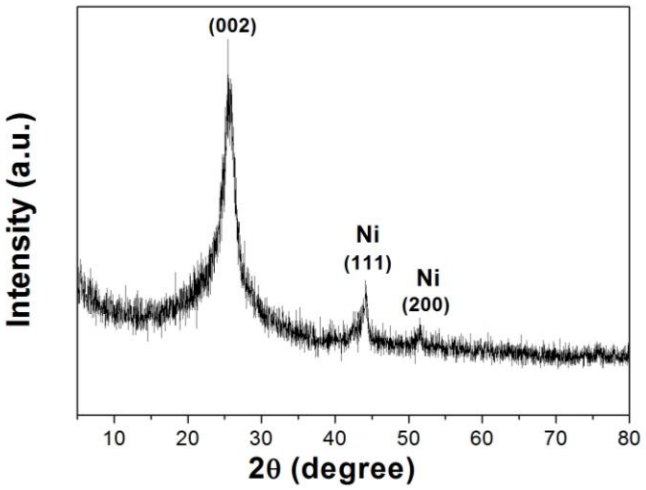

Figure S1. XRD pattern of rGN6 composite

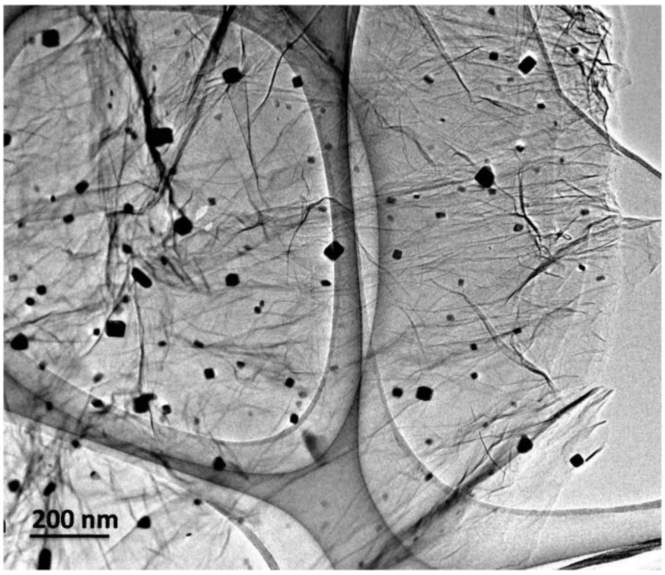

Figure S2. TEM image of rGN6 composite

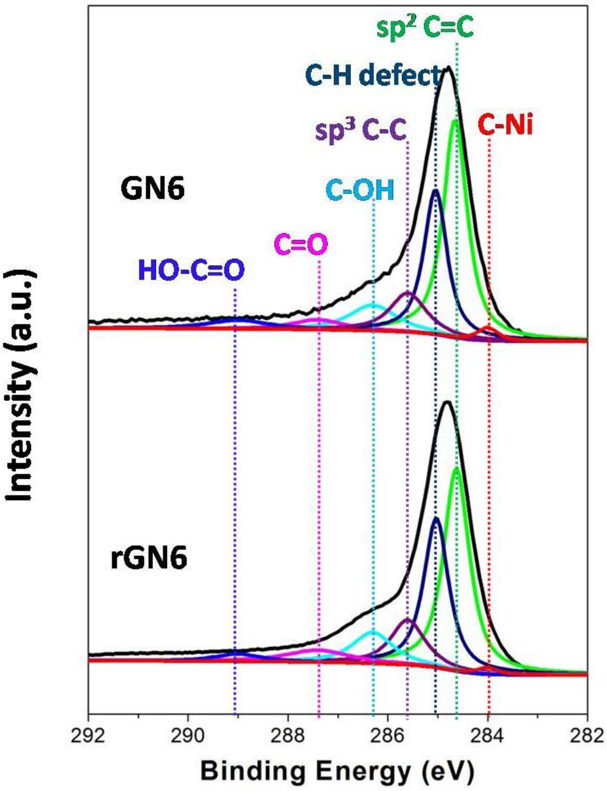

Figure S3. The XPS spectra of C 1s for GN6 and rGN6 composite.

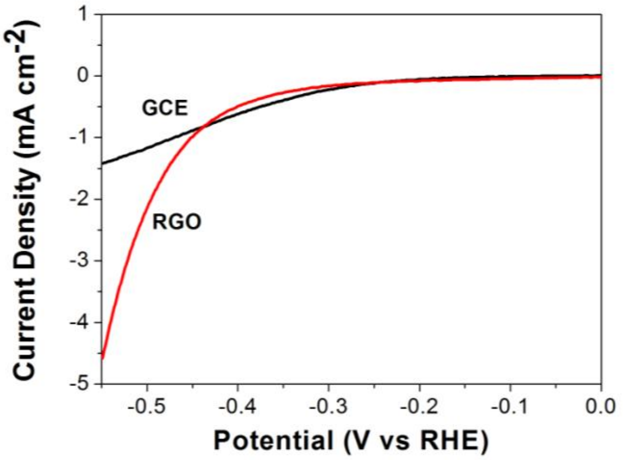

Figure S4. The LSV curves of bare glass carbon electrode (GCE) and RGO in 0.50 M H_2SO_4 solution.

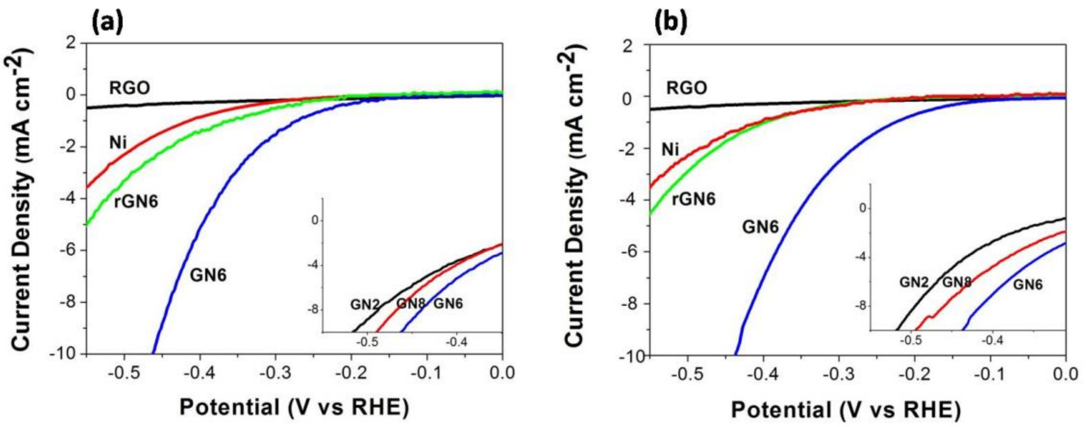

Figure S5. The LSV curves of RGO, Ni, rGN6 and GN6 in 0.50 M Na_2SO_4 solution of pH 10. (a) The as-prepared samples, and (b) The samples after 15 s soaking in 1.0 M acetate solution to remove the NiO on the surface of the Ni NPs for the composites. The insets are LSV curves of GN2, GN6, and GN8 composites.

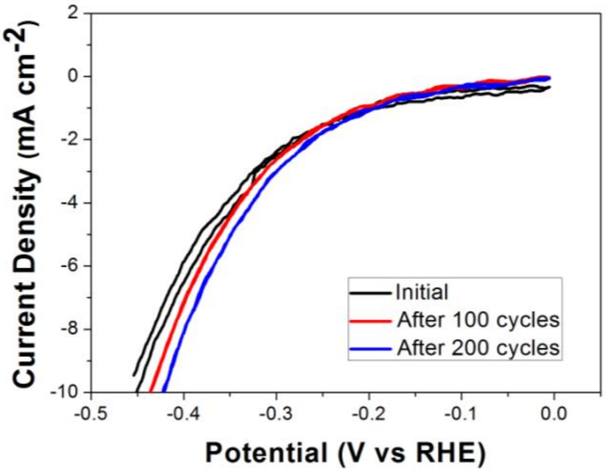

Figure S6. Cyclic voltammetry (CV) curves of GN6 composite in 0.50 M Na_2SO_4 solution of pH 10.

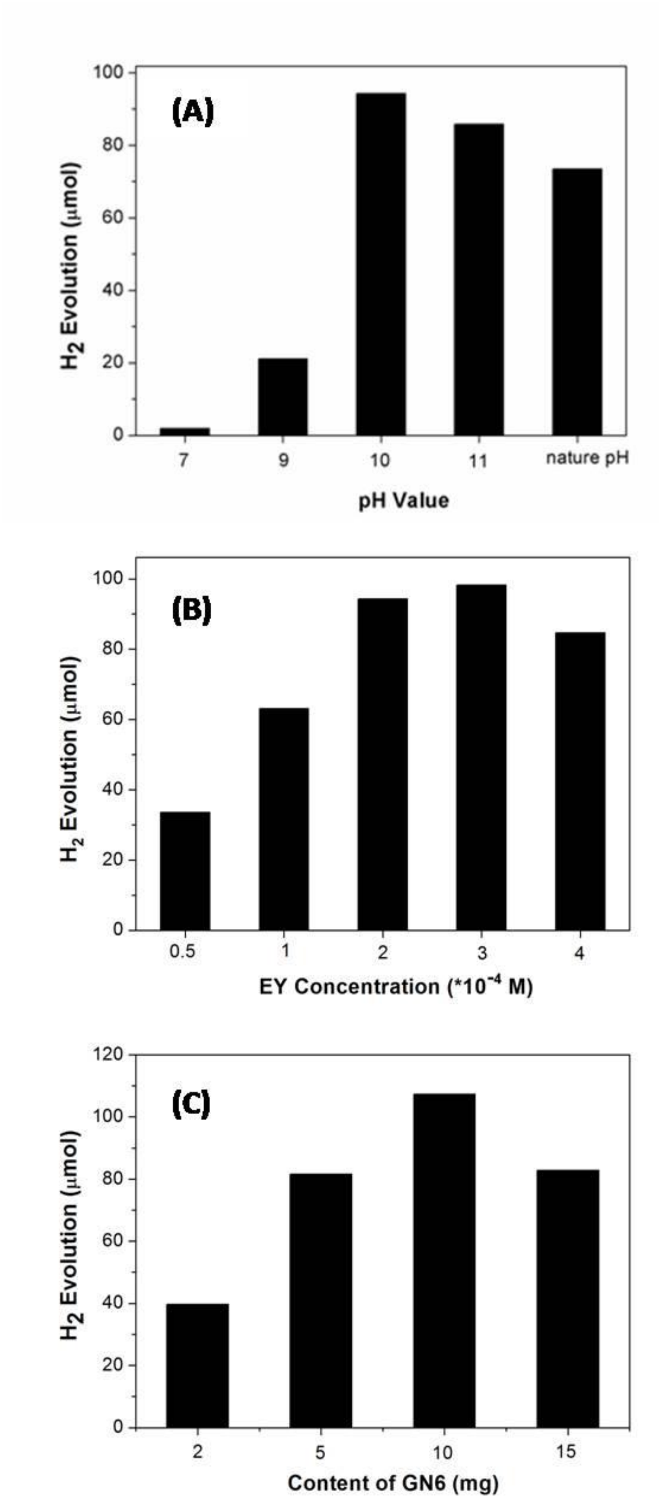

Figure S7. The effects of pH, Eosin Y (EY) concentration, and GN6 amount on photocatalytic H_2 evolution activity in EY-GN6 system. Conditions：(A) 10 mg of GN6; 2.0×10^{-4} M EY; 7.7×10^{-2} M trimethylamine (TMA); Hg lamp ($\lambda\geq420$ nm) irradiation 1 h. (B) 10 mg of GN6; 7.7×10^{-2} M TMA, pH 10; Hg lamp ($\lambda\geq420$ nm) irradiation 1 h. (C) 3.0×10^{-4} M EY; 7.7×10^{-2} M TMA, pH 10; Hg lamp ($\lambda\geq420$ nm) irradiation 1 h.

Table S1. Physical properties of RGO, GN2, GN6, GN8 and rGN6.

Sample	Ni content (wt %)	Specific surface area (m^2 g^{-1})	Crystal size of metal Ni $^{\#}$(nm)	Thickness of RGO $^{\#}$ (nm)
RGO	0	352.0	-	2.1
GN2	2.0	191.7	2.8	2.3
GN6	6.0	180.3	6.0	2.5
GN8	8.0	175.2	7.1	3.0
rGN6	6.0	50.3	17.0	3.5

$^{\#}$ The size and the thickness are calculated by the Scherrer formula using the Ni (111) and RGO (002) diffraction facet, respectively.
